# Influence of scar age, laser type and laser treatment intervals on paediatric burn scars: a systematic review and meta-analysis

**DOI:** 10.1093/burnst/tkad046

**Published:** 2024-02-02

**Authors:** Yangmyung Ma, Sabrina P Barnes, Yung-Yi Chen, Naiem Moiemen, Janet M Lord, Amanda V Sardeli

**Affiliations:** College of Medical and Dental Sciences, University of Birmingham, Edgbaston, Birmingham B15 2TT, United Kingdom; Hull York Medical School, University Rd, Heslington, York YO10 5DD, United Kingdom; College of Medical and Dental Sciences, University of Birmingham, Edgbaston, Birmingham B15 2TT, United Kingdom; College of Medical and Dental Sciences, University of Birmingham, Edgbaston, Birmingham B15 2TT, United Kingdom; Department of Burns and Plastic Surgery, Queen Elizabeth Hospital Birmingham, Mindelsohn Way, Edgbaston, Birmingham B15 2WB, United Kingdom; Scar Free Foundation Centre for Burns Research, University Hospitals Birmingham NHS Foundation Trust, Queen Elizabeth Hospital Birmingham, Mindelsohn Way, Edgbaston, Birmingham B15 2WB, United Kingdom; College of Medical and Dental Sciences, University of Birmingham, Edgbaston, Birmingham B15 2TT, United Kingdom; Scar Free Foundation Centre for Burns Research, University Hospitals Birmingham NHS Foundation Trust, Queen Elizabeth Hospital Birmingham, Mindelsohn Way, Edgbaston, Birmingham B15 2WB, United Kingdom; National Institute for Health Research Surgical Reconstruction and Microbiology Research Centre, University Hospitals Birmingham NHS Foundation Trust, Queen Elizabeth Hospital Birmingham, Mindelsohn Way, Edgbaston, Birmingham B15 2WB, United Kingdom; College of Medical and Dental Sciences, University of Birmingham, Edgbaston, Birmingham B15 2TT, United Kingdom

**Keywords:** Laser therapy, Hypertrophic burn scars, Scar outcomes, Scar age, Paediatric

## Abstract

**Background:**

Laser therapy has emerged to play a valuable role in the treatment of paediatric burn scars; however, there is heterogeneity in the literature, particularly concerning optimal timing for initiation of laser therapy. This study aims to investigate the effect of factors such as scar age, type of laser and laser treatment interval on burn scar outcomes in children by meta-analysis of previous studies.

**Methods:**

A literature search was conducted across seven databases in May 2022 to understand the effects of laser therapy on burn scar outcomes in paediatric patients by metanalysis of standardized mean difference (SMD) between pre- and post-laser intervention. Meta-analyses were performed using the Comprehensive Meta-Analysis software version 4.0. Fixed models were selected when there was no significant heterogeneity, and the random effects model was selected for analysis when significant heterogeneity was identified. For all analyses, a *p*-value < 0.05 was considered significant.

**Results:**

Seven studies were included in the meta-analysis with a total of 467 patients. Laser therapy significantly improved Vancouver Scar Scale (VSS)/Total Patient and Observer Scar Assessment Scale (Total POSAS), vascularity, pliability, pigmentation and scar height of burn scars. Significant heterogeneity was found between the studies and thus subgroup analyses were performed. Early laser therapy (<12 months post-injury) significantly improved VSS/POSAS scores compared to latent therapy (>12 months post-injury) {SMD −1.97 [95% confidence interval (CI) = −3.08; −0.87], *p* < 0.001 *vs* −0.59 [95%CI = −1.10; −0.07], *p* = 0.03} as well as vascularity {SMD −3.95 [95%CI = −4.38; −3.53], *p* < 0.001 *vs* −0.48 [95%CI = −0.66; −0.30], *p* < 0.001}. Non-ablative laser was most effective, significantly reducing VSS/POSAS, vascularity, pliability and scar height outcomes compared to ablative, pulse dye laser and a combination of ablative and pulse dye laser. Shorter treatment intervals of <4 weeks significantly reduced VSS/POSAS and scar height outcomes compared to intervals of 4 to 6 weeks.

**Conclusions:**

Efficacy of laser therapy in the paediatric population is influenced by scar age, type of laser and interval between laser therapy application. The result of this study particularly challenges the currently accepted initiation time for laser treatment. Significant heterogeneity was observed within the studies, which suggests the need to explore other confounding factors influencing burn scar outcomes after laser therapy.

Highlights

- This study is the first to examine the effects of laser therapy on the paediatric population through meta-analysis.
- This study showed that early initiation (<12 months) of laser therapy can be effective in management of hypertrophic burn scars.
- The currently accepted initiation time for laser treatment must be reconsidered.

## Background

Burn scars can have a significant effect on the quality of life of a child. These scars are often accompanied by complications such as contraction, pain, pruritus, erythema and limited mobility, which can hamper a child’s physical, psychological and social wellbeing, as well as their family’s wellbeing [1,2]. Due to the high scar prevalence in this population and the long-term impact of complications, investigating outcomes of treatment and rehabilitation is important [2–4]. Several methods of management for hypertrophic burn scars currently exist but none have been found to be completely effective. However, laser therapy has emerged as playing a valuable role as an adjunctive or definitive form of therapy for paediatric burn scars in recent times due to it being minimally invasive, low risk and reducing the post-operative recovery period.

The lasers used are classified into ablative lasers, non-ablative lasers and pulse dye lasers (PDLs). Ablative CO_2_ lasers are often used to target both dermal and epidermal layers of the skin for collagen remodelling through a photothermal effect, whereas non-ablative lasers are non-selective in their target and are known to improve scar thickness and volume by restoring damaged collagen without injuring or removing the epidermis [5]. PDLs work by using selective thermolysis to target superficial blood vessels. They deliver pulsed laser energy at a lower wave light frequency, which is primarily absorbed by oxyhaemoglobin, which subsequently destroys superficial vessels [6]. Despite the important role of lasers in burn scar management, heterogeneity regarding the efficacy of this modality of treatment exists in the literature, which may be dependent upon the type of laser used, wavelength of laser and the optimal timing for commencing laser intervention [5,7].

Scar maturation and characteristics such as patient age, skin type, type of scar and comorbidities are important factors in the decision to initiate laser therapy. This has led to heterogeneity in the literature, particularly surrounding optimal timing for initiation of laser therapy and in the outcomes of the treatment [8]. Optimal time to initiate laser intervention is often considered to be when the scar has fully matured, with currently accepted treatment parameters suggesting waiting for 1 year following burn injuries [9]. However, recent studies have reported the benefit of early initiation of laser therapy, with a decrease in complications such as contractures, improvement in mobility and improvement in the overall rehabilitation process [10,11]. Furthermore, with research also suggesting no association between the incidence of complications of laser treatments and scar age at time of treatment, early laser treatment has become a potential method to reduce scar formation [8].

Recent meta-analyses have demonstrated the effectiveness of laser therapy in patients with hypertrophic burn scars [12–15]. However, the studies have only focused on the use of one laser in the adult population and observed significant heterogeneity in the data. No meta-analysis to date has considered the effects of laser therapy on burn scar outcomes in the paediatric population or the effects of optimal timing of laser therapy (scar age) in this age group.

The aim of this study therefore was to identify the effect of laser therapy on burn scar outcomes [Vancouver Scar Scale (VSS)/Total Patient and Observer Scar Assessment Scale (POSAS) scores, vascularity, pliability, pigmentation and scar height] through a comprehensive meta-analysis. The effect of different times to initiate treatment (scar age), type of laser used, laser treatment interval and complications with laser therapy were considered. This study focuses on the paediatric population only, due to different pathophysiological responses to burn injuries as well as different responses to laser therapy between adult and paediatric populations [16,17].

## Methods

This review was reported according to the Preferred Reporting Items for Systematic Review and Meta-Analyses (PRISMA) guidelines.

### Protocol and registration

The study protocol was registered with PROSPERO (CRD42022347836).

### Eligibility criteria

The Population, Intervention, Comparison, Outcome, Study design (PICOS) inclusion criteria were: (1) human paediatric patients (<18 years of age) with any post-burn hypertrophic scars; (2) undergoing laser therapy; (3) assessing subjective VSS/Total POSAS scores for vascularity, pliability, pigmentation, scar height and/or objective scar measurement tools (e.g. via ultrasound guided measurement); (4) in retrospective, prospective studies or randomized control trials (RCTs). Only studies written in English or Chinese were included. There was no date of publication restriction.

### Exclusion criteria

The exclusion criteria for this study included: acne scars, surgical scars, conference abstracts, adult studies (≥18 years old), article reviews, literature reviews, case reports and animal studies.

### Information sources

The following databases were accessed for the literature search: MEDLINE (PubMed), Google Scholar, EMBASE, Scopus, Cochrane Database of Systematic Reviews and University Library of York and Hull. All databases were accessed from 25 May 2022 to database inception. Forward and backward citation searching as well as grey literature was checked to identify further articles.

### Search

The search strategy involved using pre-defined keywords with corresponding medical subject headings which included ‘hypertrophic scar’, ‘cicatrix’, ‘keloid’, ‘scar’, ‘burn’, ‘major burn’, ‘thermal injury’, ‘severe burn’, ‘laser’, ‘laser therapy’, ‘ablative’, ‘pulse dye laser’, ‘ablation therapy’.

### Study selection

All articles were downloaded onto Covidence, a screening and data extraction programme. Duplicates were removed and remaining articles were screened by two authors independently. Articles were included or excluded using the aforementioned criteria. Any discrepancies concerning an article’s inclusion/exclusion were resolved through analysing the full text and through discussion with all authors. Articles in Chinese were translated into English.

### Data collection process

Data extraction was conducted by using a bespoke data extraction form. Data was extracted for the following categories: population (number of patients, age, scar age), intervention (time of initiation of treatment, laser type, number of treatments, treatment interval, time of assessment, scar assessment tools used) and outcomes of the study (overall VSS/Total POSAS scores, vascularity, pliability, pigmentation, scar height, complications). Two independent reviewers extracted the mean, standard deviation and sample size of each outcome before and after laser interventions for meta-analyses.

‘Laser’ was defined as scar therapy utilizing photothermal energy to target intra- and extra-cellular structures within the scar tissue [18]. ‘Hypertrophic burn scars’ were defined as pathological scarring due to major burns characterized by red, raised and rigid scar tissue that contracts and limits normal motion of the skin [19]. Scar age was categorized into ‘early’ or ‘latent’, with ‘early’ being ≤12 months old and ‘latent’ being >12 months old, based on the currently accepted treatment parameters of waiting 1 year following burn injury [9]. All patients <18 years old were considered paediatric.

### Risk of bias in individual studies

To determine the methodological quality and risk of bias of the included articles, full-text articles were assessed using the ROBINS-E tool for non-randomized studies of interventions and RoB tool for randomized controlled trials [20,21]. The results are presented in Robvis format.

### Statistical analysis

Five meta-analyses were performed using the Comprehensive Meta-Analysis (CMA) software version 4.0, testing the effects of early and latent laser therapy using (1) overall scar improvement (assessed by VSS and Total POSAS in score points), (2) scar vascularity (score points), (3) scar pliability (score points), (4) scar pigmentation (score points) and (5) scar height (score points/nanometres) in burn scars of paediatric patients. Effect size was calculated based on the standard mean difference between before and after intervention (retrospective or prospective studies). Fixed models were selected when there was no significant heterogeneity, and the random effects model was selected for analysis when significant heterogeneity was identified. Conservative pre–post correlations of 0.05 were assumed [22].

To explore confounding factors that could be contributing to the heterogeneity in data, subgroup analyses were performed. The following subgroups were tested: scar age [early (<12 months) and latent (>12 months) initiation of treatment], type of laser (ablative, PDL, non-ablative, PDL and ablative combined), interval length of laser treatment application (<4 weeks, 4–6 weeks and 6–8 weeks) and complications reported [presence (bleeding, swelling, hyperpigmentation, hypopigmentation, pain, blisters, pruritus, erythema, seepage, etc.) and absence (no complications)]. When an included study did not fit the category of subgroup or did not report the information, the study was excluded from that specific subgroup analysis. For all analyses, a *p*-value < 0.05 was considered significant. The Egger test was used to test the publication bias considering a *p*-value ≤ 0.05.

## Results

**Figure 1:**
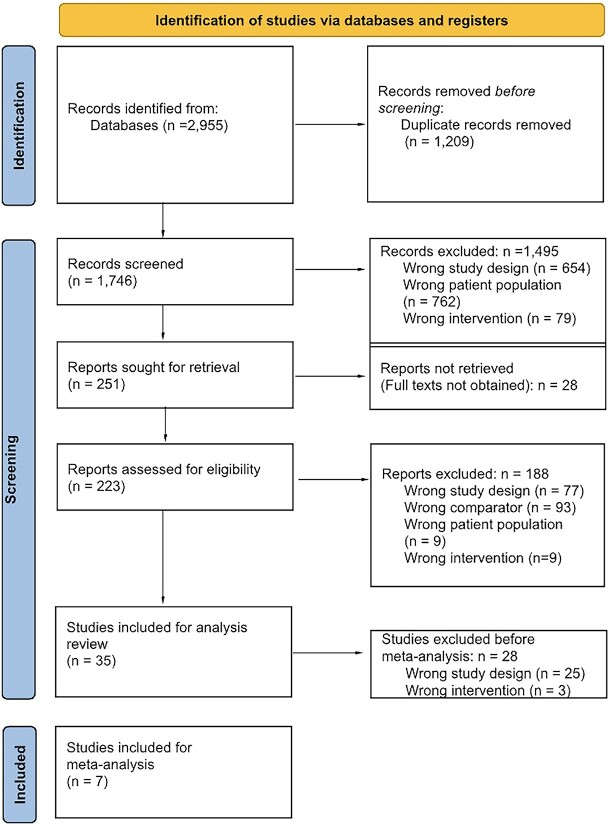
Flowchart of selection of studies

The initial search yielded 2955 studies that were subject to the inclusion and exclusion criteria, leading to 7 studies that were used for meta-analyses. Figure 1 presents this data via a PRISMA flow diagram.

### Characteristics of the studies considered for use in analysis

The 7 studies included in the meta-analysis had publication dates from 2012 to 2021. Two RCTs, three prospective studies and two retrospective studies were found [23–29]. A total of 467 participants were included, with the largest population size in a single study of 165 [28]. The studies were undertaken in five countries, with the USA being the most common location. Average patient age was 10 years (range: 3–17) with a male to female ratio of 1 : 1. Ablative CO_2_ lasers were most used in three studies at a frequency of 10,600 nm. PDL (595 nm) was used in one study, non-ablative laser (Nd:YAG laser 1064 nm) in one study and a combination of PDL and ablative in two studies. Treatment duration, treatment interval and number of sessions varied between the studies. The studies mostly relied on VSS as an outcome measure. One ‘minor’ case of complications was reported in the studies, with a majority not reporting any complications. Table 1 shows the characteristics of the included studies.

**Table 1:** Characteristics of the studies included.

**Author,** **publication date (ref)**	**Country**	**No. of patients**	**Mean age/range** **(years)**	**Scar age category: range (months)**	**Laser type** **(wavelength, nm)**	**Total no. of sessions (interval, weeks)**	**Time of assessment**	**Measurement tools used**	**Outcomes reported**	**Complications**
Bailey 2012 [23]	USA	13	15.2	Latent: 60–120	PDL (595)	>2 (6)	Every 6 weeks	VSS, volumetric measure, digital pictures	SEry, SH, SElas	NR
Khandelwal 2014 [24]	USA	40	18	Latent: 60–120	Ablative (10,600)	2–4 (NR)	NR	VSS	OSI, Vas, Pigm, Pli, SH	None
Zuccaro 2018 [25]	Canada	125	6.62	Early and latent: 6–60	PDL + ablative (595, 10 600)	NR (4.8)	NR	VSS, Specific scale	OSI, Vas, Pigm, Pli, SH, Pru	Minor
Patel 2019 [26]	USA	49	4.86	Latent: 24–60	Ablative (10,600)	Average 3.88 (4–8)	NR	POSAS	OSI, Pigm, Vas, SH, STex	None
Khedr 2020 [27]	Egypt	50	16.2 ± 5.92	Early: 6–12	Non-ablative (1064)	4–10 (4)	At 1, 3, 12 months	VSS, Biopsy	OSI, Vas, Pigm, Pli, SH	None
Li 2021 [28]	China	165	3.5 ± 3.02	Early: 6–12	Ablative (10,600)	2–6 (NR)	NR	VSS, Ultrasound	OSI, Vas, Pigm, Pli, SH, STex, Pain, Pru, BP	NR
Matuszczak 2021 [29]	Poland	25	6.4	Early: 6–12	PDL + ablative (595, 10,600)	2 (6–8)	At 0, 1 months	VSS, POSAS, Volumetric measure	OSI, Vas, Pigm, Pli, SH, STex, Pain, Pru, CL	NR

*POSAS* Patient and Observer Scar Assessment Scale, *PDL* pulse dye l*aser*, *VSS* Vancouver Scar Scale, *OSI* overall scar improvement, *Pigm* pigmentation, *Pli* pliability, *Vas* vascularity, *SH* scar height, *STex* scar texture, *Pru* pruritus, *SEry* scar erythema, *SElas* scar elasticity, *CL* collagen levels, *BP* blood perfusion, *NR* not reported

### Quality of studies

**Figure 2:**
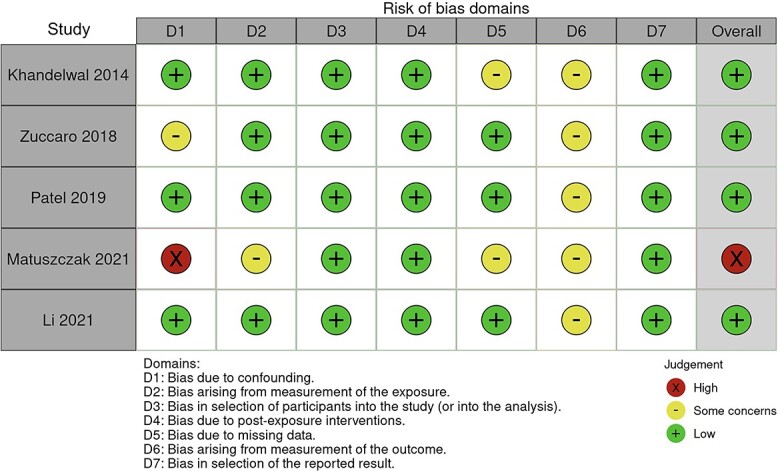
Robvis—ROBINS-E assessment of bias for non-randomized studies

**Figure 3:**
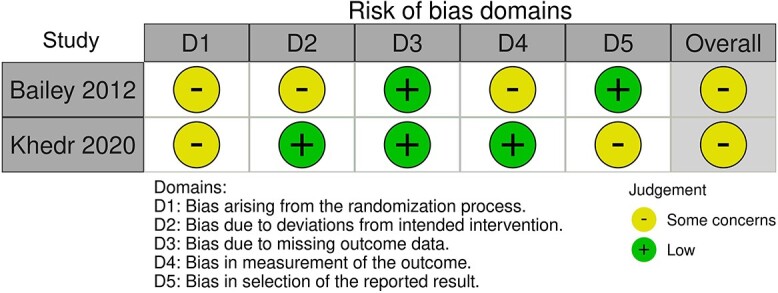
Robvis—RoB assessment of bias for randomized studies

Four of the non-randomized studies scored an overall low risk of bias [24–26,28]. Most studies had some concerns with bias arising from measurement of the outcome. One non-randomized study scored an overall high risk of bias due to a high risk of counfounding bias [29]. Two RCTs showed some risk of bias overall, with bias arising from the randomization process [23,27]. Figures 2 and 3 shows the risk of bias assessment for non-randomized studies and randomized studies respectively.

### Evidence synthesis

**Figure 4:**
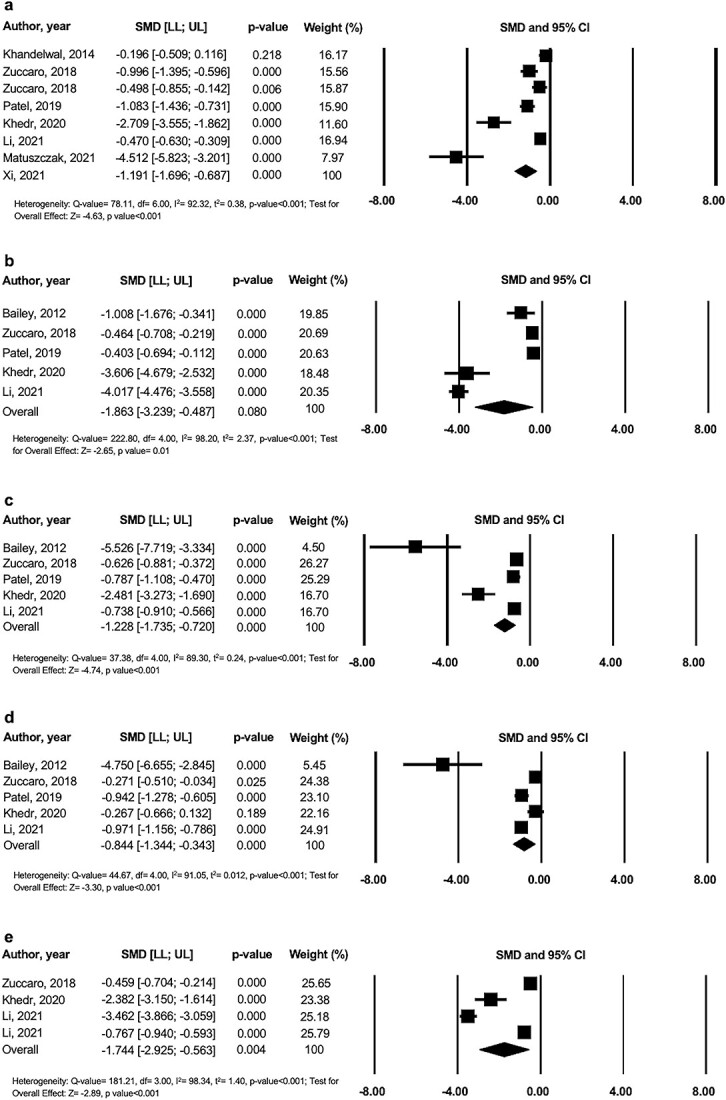
Forest plots of the effect of laser therapy on (**a**) VSS/POSAS, (**b**) vascularity, (**c**) pliability, (**d**) pigmentation and (**e**) scar height. *CI* confidence interval, *LL* lower limit, *POSAS* Patient and Observer Scar Assessment Scale, *SMD* standard mean difference, *UL* upper limit, *VSS* Vancouver Scar Scale

The results of this study showed that laser therapy significantly reduced VSS/Total POSAS scores (Figure 4a), vascularity (Figure 4b), pliability (Figure 4c), pigmentation (Figure 4d) and scar height (Figure 4e) in the overall analyses. No risk of publication bias for vascularity, pigmentation and scar height meta-analyses were found (*p*-value of Egger test > 0.05). However, there was significant risk of publication bias for the VSS/POSAS and pliability meta-analyses (*p*-value of Egger test ≤ 0.05).

Table 2 shows the subgroup analyses for the outcomes tested. Due to the lack of comparable data available with regards to patients with or without complications, as well as the total number of laser therapy sessions involved, these specific subgroup analyses were not performed.

**Table 2:** Subgroup analysis of effect of laser therapy of hypertrophic scars on (A) VSS/POSAS, (B) vascularity, (C) pliability, (D) pigmentation and (E) scar height.

**Subgroup**	**K**	**Study (reference)**	**SMD**	**LL and UL of 95% CI**	***P* value within**	***P* value between**
**A. VSS/POSAS total scores**						
**Scar age**						
Early	4	(25,27,28,29)	-1.97	[−3.08 to −0.87]	<0.001	0.03
Latent	3	(24,25,26)	-0.59	[−1.10 to −0.07]	0.03	
**Laser type**						
Ablative	2	(24,26)	-0.64	[−1.51 to 0.23]	0.15	<0.001
PDL	1	(28)	-0.47	[−0.63 to −0.31]	<0.001	
Non-ablative	1	(27)	-2.71	[−3.56 to −1.86]	<0.001	
PDL + ablative	3	(25,25,29)	-1.75	[−3.00 to −0.50]	0.01	
**Interval length (weeks)**						
<4	2	(26,27)	-1.85	[−3.44 to −0.26]	0.02	<0.001
4–6	2	(25,25)	-0.74	[−1.23 to −0.25]	<0.001	
6–8	1	(29)	-4.51	[−5.82 to −3.20]	<0.001	
**B. Vascularity**						
**Scar age**						
Early	2	(27,28)	-3.95	[−4.38 to −3.53]	<0.001	<0.001
Latent	3	(23,25,26)	-0.48	[−0.66 to −0.30]	<0.001	
**Laser type**						
Ablative	1	(26)	-0.40	[−0.69 to −0.11]	0.01	<0.001
PDL	2	(23,28)	-2.52	[−5.47 to 0.43]	0.09	
Non-ablative	1	(27)	-3.61	[−4.68 to −2.53]	<0.001	
PDL + ablative	1	(25)	-0.46	[−0.71 to −0.22]	<0.001	
**Interval length (weeks)**						
<4	2	(26,27)	-1.96	[−5.10 to 1.18]	0.22	0.42
4–6	2	(23,25)	-0.64	[−1.15 to −0.14]	0.01	
**C. Pliability**						
**Scar age**						
Early	2	(27,28)	-1.56	[−3.27 to 0.14]	0.07	0.72
Latent	3	(23,25,26)	-0.73	[−0.93 to −0.53]	<0.001	
**Laser type**						
Ablative	1	(26)	-0.79	[−1.11 to −0.47]	<0.001	<0.001
PDL	2	(23,28)	-3.00	[−7.69 to 1.68]	0.21	
Non-ablative	1	(27)	-2.48	[−3.27 to −1.69]	<0.001	
PDL + ablative	1	(25)	-0.63	[−0.88 to −0.37]	<0.001	
**Interval length (weeks)**						
<4	2	(26,27)	-1.59	[−3.25 to 0.06]	0.06	0.60
4–6	2	(23,25)	-2.95	[−7.75 to 1.85]	0.23	
**D. Pigmentation**						
**Scar age**						
Early	2	(27,28)	-0.64	[−1.33 to 0.05]	0.07	0.29
Latent	3	(23,25,26)	-1.29	[−2.27 to −0.30]	0.01	
**Laser type**						
Ablative	1	(26)	-0.94	[−1.28 to −0.61]	<0.001	0.12
PDL	2	(23,28)	-2.74	[−6.43 to 0.96]	0.15	
Non-ablative	1	(27)	-0.27	[−0.67 to 0.13]	0.19	
PDL + ablative	1	(25)	-0.27	[−0.51 to −0.03]	0.03	
**Interval length (weeks)**						
<4	2	(26,27)	-0.61	[−1.27 to 0.05]	0.07	0.43
4–6	4	(23,25)	-2.41	[−6.79 to 1.98]	0.28	
**E. Scar height**						
**Scar age**						
Early	3	(27,28,28)	-2.20	[−4.18 to −0.22]	0.03	0.09
Latent	1	(25)	-0.46	[−0.70 to −0.21]	<0.001	
**Laser type**						
PDL	2	(28,28)	-2.11	[−4.75 to 0.53]	0.12	<0.001
Non-ablative	1	(27)	-2.38	[−3.15 to −1.61]	<0.001	
PDL + ablative	1	(25)	-0.46	[−0.70 to −0.21]	<0.001	
**Interval length (weeks)**						
<4	1	(27)	-2.38	[−3.15 to −1.61]	<0.001	<0.001
4–6	1	(25)	-0.46	[−0.70 to −0.21]	<0.001	

*CI* confidence interval, *LL*  lower limit, *UL*  upper limit, *PDL*  pulse dye laser, *POSAS* Patient and Observer Scar Assessment Scale, *SMD* standardized mean difference, *VSS* Vancouver Scar Scale, *K* number of studies

Early laser therapy led to significantly higher improvement in VSS/Total POSAS scores (1.4-point difference, *p* < 0.03) and vascularity (3.5-point difference, *p* < 0.001) compared to latent laser therapy. However, it is noteworthy that latent laser intervention significantly improved all five burn scar outcomes in the paediatric population.

Non-ablative laser was the most effective type of laser by significantly reducing VSS/Total POSAS (*p* < 0.001), vascularity (*p* < 0.001), pliability (*p* < 0.001) and scar height outcomes (*p* < 0.001). This was followed by PDL + ablative for VSS/Total POSAS (*p* = 0.01), vascularity (*p* < 0.001) and scar height outcomes (*p* < 0.001) and ablative laser for the pliability outcome (*p* < 0.001).

Significant differences between results for laser intervals were only found for the VSS/Total POSAS and scar height outcomes. For VSS/Total POSAS, an improved response was seen for treatment interval lengths of 6–8 weeks followed by <4 weeks and 4–6 weeks. For scar height, a greater reduction in scores was observed when lasers were used at shorter intervals of <4 weeks compared to 4–6 weeks.

Sensitivity analysis of raw mean difference (RMD) for each scale of each outcome was performed to infer the clinical significance of these results. Laser therapy reduced 3.07 points from VSS scale 0–13 ([–4.66; –1.48], *p* < 0.001, number of studies K = 6) while a reduction of 1.79 ([−2.25; −1.33], *p* < 0.001, K = 1) was observed for Total POSAS. For vascularity outcome, RMD for VSS points 0–3 was −1.22 ([−1.82; −0.62], *p* < 0.001, K = 4) and −0.96 ([−1.63; −0.29], *p* < 0.001, K = 1) for Total POSAS. Pliability RMD showed a reduction of 1.04 for VSS points 0–5 ([–1.69; –0.40], *p* < 0.001, K = 4) and a reduction of 1.78 ([−2.41; −1.15], *p* < 0.001, K = 1) for Total POSAS. RMD for pigmentation for VSS points 0–2 was −0.79 ([−1.48; −0.11], *p* = 0.02, K = 4) and − 1.65 ([−2.14; −1.16], *p* < 0.001, K = 1) for Total POSAS. For scar height, laser therapy reduced 0.71 points from VSS scale 0–3 ([−1.16; −0.27], *p* < 0.001, K = 3) with a reduction of 1.49 mm ([−1.56; −1.42], *p* < 0.001, K = 1) via ultrasound.

## Discussion

Though the molecular and cellular basis of hypertrophic burn scar formation is well understood, the mechanisms underpinning scar reduction induced by laser therapy are not fully understood [15]. The theory of Phototherapy on burn scars however relies on the controlled formation of new collagen by causing either a photochemical reaction or heating to scars that have formed due to abnormal healing processes involving increased collagen and fibronectin synthesis, fibroblast proliferation and neovascularization [5,7]. It is perhaps this paucity of understanding with regard to the exact processes by which phototherapy reduces burn scars that has led to several studies focussing on the various effects of laser type, duration and optimal timing in an attempt to reduce heterogeneity in outcomes [30,31]. This is particularly pertinent in the paediatric population where burn injuries are highly prevalent and where patients undergo different pathophysiological processes to adult burns patients [17,28]. The aim of this meta-analysis was to address this heterogeneity by considering variables such as timing of treatment after injury, laser type and intervals for laser intervention in the paediatric population to aid clinicians and patients in making evidence-based decisions when laser therapy is opted for as a method for burn scar management.

In this analysis, seven studies involving 467 patients were found for inclusion. The findings showed that laser therapy is an efficient method of treatment for hypertrophic burn scars for paediatric patients by improving burn scar features. VSS/Total POSAS scores particularly improved when laser therapy was used before 12 months since injury and through the use of non-ablative lasers.

Wound healing typically occurs in four discrete phases, inflammation, proliferation, remodelling and maturation [32]. The inflammatory phase for example involves the release of cytokines and chemokines as well as the recruitment of fibroblasts and macrophages to restore the skin barrier. The proliferation stage involves the replacement of the provisional wound matrix with granulation tissue and collagen bundles; this stage can continue for up to 6 weeks [33]. The remodelling phase involves the differentiation of fibroblasts into myofibroblasts that contract and reduce the size of the wound before entering the maturation phase [32]. The disturbance of normal collagen production and collagenase synthesis, particularly during the maturation phase, leads to disorganized bundles of collagen that are cross-linked tightly, creating a hypertrophic scar [34]. Targeting this process of disorganized growth in the early stages of wound healing has therefore become a recent topic of interest. For example, a RCT in 2019 showed positive results for laser therapy in adults at <3 months from injury by significantly decreasing scar formation compared to untreated areas on the same wounds. The study observed an improvement in the Manchester Scar Scale and upon blinded evaluation of photographs of the scars. Histology and optical coherence tomography also demonstrated re-organization of the skin structure in scars caused by moderate-to-severe burn injuries [9]. These results have challenged the currently accepted treatment parameters of waiting 1 year following burn injury before laser therapy and have provided a new time course to treat severe burn and trauma injuries in this way [9].

The present study found significant reduction particularly in VSS/Total POSAS scores with early laser therapy. This may be attributed to evidence which has suggested that hypertrophic scars take significantly less time to completely mature in the paediatric population [35,36]. For example, a prospective study in 2017 showed the importance of time-to-healing in preventing hypertrophic scarring in paediatric burn patients [35]. In patients who took <21 days to heal, 8.1% of wounds developed hypertrophic scarring compared to 56% of patients who took >21 days to heal. Although the inclusion criteria for Chipp et al.’s study did not involve patients who underwent laser intervention, this study suggests that maturation of burn scars could occur as early as 3 weeks and thus early intervention to target the earlier phases of wound healing may be necessary for the prevention of hypertrophic scar formation. Furthermore, a prospective study of burn scar maturation in paediatric patients showed a rapid peak of scarring of 1–2 months and scar maturation of 9–13 months for patients <18 years old [36]. Although this paper did not investigate scar maturation after laser therapy, their data suggest that the hypertrophic burn scars that were considered as early phase in our analysis were treated within the first three phases of wound healing and the potential for disorganized growth within these phases was prevented.

The subgroup analysis also showed that laser type had a significant impact on the main results, with non-ablative lasers showing the greatest effect in significantly reducing VSS/Total POSAS, vascularity, pliability and scar height outcomes. Selection of laser type depends on the principle that the targeted tissue has a greater optical absorption at a specific wavelength compared to the surrounding tissue [5,7]. Non-ablative lasers typically spare the epidermis and selectively damage the dermis, which results in less superficial damage compared to ablative lasers [5]. Non-ablative lasers have also been known to help reduce vascularity by reducing erythema, pruritis, pigmentation and hypertrophy and can therefore be useful in the early stages of wound healing when the hypertrophic scar is thinner and more vascular, as found in a previous study [37]. A retrospective study in 2012 highlighted the effectiveness of non-ablative lasers in patients with burn scars, with overall improvement in 90% of subjects and a majority of patients with improved skin texture, dyschromia and hypertrophy/atrophy [38]. However, this study focused on the adult population and the wavelength of the non-ablative laser used was different to that found in the present study. Similarly, a RCT in 2015 supported the effectiveness of non-ablative lasers by significantly improving burn scar appearance, which was confirmed by histological evidence of collagen remodelling at 6 months [39]. Although the study by Taudorf et al focused on non-ablative laser therapy on mature burn scars and did not involve paediatric patients, due to the particular effectiveness of this laser in inducing histological improvement even after maturation, it again demonstrates the potential benefit in improving burn scar outcomes if utilized before scar maturation.

Significant reduction was observed only for VSS/Total POSAS and scar height outcomes, where scars that were treated at <4 week intervals had improved scar outcomes compared with those treated at 4–6 week intervals. Scar recurrence is a major issue with pathological keloid and hypertrophic burn scars, with recurrence reported to present as early as 2 weeks and up to 3 years following ablative laser therapy [40,41]. Studies that utilized lasers at shorter intervals may have observed improved outcomes due to initiating treatment before the cellular and molecular processes of scar recurrence could occur.

Finally, we wanted to investigate whether any complications, such as blistering, bleeding etc. affected the main results. However, all studies either did not report any complications, had no complications or reported ‘minor’ complications that were resolved spontaneously. This suggests that laser therapy can be a safe method of treatment in the paediatric population.

The main limitations in this meta-analysis were the significant study heterogeneity and the small number of studies analysed. This analysis addressed some of the confounding factors that could have influenced this heterogeneity, but other factors such as patient age, sex, skin type and location of the burn scar, that could also explain this heterogeneity, were not considered as they were not differentiated within each study. Of note, total number of sessions was an important confounding factor that was not analysed in this study. This was due to the majority of data being presented as a range by the individual studies, thus preventing comparability of results and introducing method bias. Furthermore, the small number of studies affected the reliability of subgroup analysis as some of the results of the subgroup analysis were based on one study. The small number of studies in subgroup analysis also prevented further analysis of the data to isolate outcomes belonging to a specific subgroup within another subgroup. Subgroup analysis is by nature an exploratory analysis with a low level of evidence, since it is based on comparisons of the different studies, and thus the results of such analysis should be interpreted with caution. However, our fundings give grounds for further studies to be conducted and possibly to confirm the specific hypotheses raised within the subgroup analyses. Finally, there was a lack of controls in this study, making it difficult to ascertain whether scar improvement was solely due to the laser therapy or due to the expected improvement in scar formation with time.

## Conclusions

Early initiation of laser therapy can be effective in the management of hypertrophic burns scars in paediatric patients through improvement of some of the burn scar outcomes. This perhaps suggests that the currently accepted initiation time for laser treatment should be re-considered. The type of laser and interval length of laser therapy sessions influences effectiveness whereby studies that used non-ablative lasers at shorter treatment intervals observed the greatest improvement of burn scar outcomes.

## Data Availability

The datasets used and/or analysed during the current study are available from the corresponding author upon reasonable request.

## Abbreviations

PDL: Pulse dye laser; POSAS: Patient and Observer Scar Assessment Scale; PRISMA: Preferred Reporting Items for Systematic Review and Meta-Analyses; RCT: Randomized control trial; RMD: Raw mean difference; SMD: Standardized mean difference; VSS: Vancouver Scar Scale.
